# Measurement error is lower for visual analogue scales than for slider scales

**DOI:** 10.3758/s13428-026-03108-8

**Published:** 2026-07-22

**Authors:** Tim Angelike, Ulf-Dietrich Reips

**Affiliations:** 1 Research Methods, Assessment, and iScience, Department of Psychology, University of Konstanz, Fach 31, Konstanz, Germany; 2 Department of Psychology, Philipps-Universität Marburg, Marburg, Germany

**Keywords:** Measurement error, Formatting error, Rating scale, Visual analogue scale, Slider scales, Data quality, Web survey

## Abstract

Errors in measurement can arise in study and survey responses when there is a discrepancy between the intended and selected response. A significant portion of the scientific discourse has centered on the comparison of discrete and continuous response scales. In this study, we examined various continuous rating scales to ascertain which scale would yield the lowest measurement error. To this end, we compared visual analogue scales (VAS) with different slider scales in an online study (*N* = 222) that built upon the original work by Reips and Funke (2008). In this study, participants were asked to estimate where a percentage value would lie on a line ranging from 0 to 100%. The slider scale conditions differed in the initialization of the slider thumb (left, middle, right), with an additional slider condition that employed the default HTML slider with a large thumb without any modifications. The findings of this study suggest that the measurement error in estimating the true value was lower in VAS than in slider scales. Additionally, our findings suggest that VAS lead to reduced variability in errors within and between individuals. Contrary to our initial hypothesis, we did not find a higher measurement error in the default slider than in the other slider conditions. The findings of this study suggest that VAS may offer a more precise means to the assessment of ratings. Practitioners who intend to employ continuous rating scales should consider employing VAS or at least carefully consider the pitfalls of slider scales.

The goal of this paper is to conduct a systematic comparison of the various types of continuous rating scales commonly utilized in survey methodology, as well as in psychological and medical research: visual analogue scales (VAS) and slider scales. To this end, a replication of Reips and Funke (2008) was conducted in an online study to assess which type of continuous rating scale would produce the lowest measurement error. In the study, participants were tasked with estimating the location of various percentages on a line ranging from 0 to 100%. It is important to note that many studies will not require participants to provide explicit percentage ratings, e.g., when responding to subscales of a Big Five personality inventory or related constructs (see for example, Kuhlmann et al., 2017). However, the present study focuses on the measurement error that occurs when participants map their internal response to an item onto the rating scale (see also the *question–answer process* by Schwarz & Oysermann, 2001). In response to a personality inventory item, respondents must comprehend the item content, assess their inner state, and formulate an internal response to the question before mapping their response to the available scale. Thus, it is impossible to isolate these behaviors from measurement per se (see e.g. Narens & Skyrms, 2020). In replicating Reips and Funke (2008), the direct inquiry of percentage ratings reduces the error of measurement associated with the response processes that take place before indicating the answer to the item. This enables a more accurate study of the influence of using different rating scales. A variety of slider scales were utilized, with the initialization of these scales occurring at either the left, center, or right edge of the rating scale (Fig. 1). The findings of this study suggest that VAS yielded lower measurement error than slider scales. Furthermore, the variability in the measurement error within and between individuals was also lowest under VAS.

Accurate measurement of participant responses is imperative for any study that involves the expression of agreement with a statement or any kind of continuous judgment rating, whether conducted in a laboratory or online setting. Employing an appropriate scale is also important when inquiring about participants’ personalities. Although an objectively true value may be difficult to determine, participants must still map their internal responses to the question at hand onto a response scale (Schwarz & Oyserman, 2001). The response scale should therefore enable respondents to express their answers with minimal information loss. A significant portion of the scientific discourse has centered on the comparison of discrete rating and continuous rating scales. The Likert scale (Likert, 1932) is arguably the most prevalent and renowned rating scale. It instructs participants to indicate their degree of agreement with a statement on a discrete scale, often ranging from 1 to 5 or 1 to 7, with options from “completely disagree” to “completely agree.” However, the precise optimal number of discrete rating options is a subject of considerable controversy (Abulela & Khalaf, 2024; Wu & Leung, 2017). Conversely, continuous rating scales do not divide the response scales into discrete categories, thereby enabling participants to offer responses of a more nuanced nature. One frequently employed rating scale utilized to obtain continuous ratings is VAS (originally proposed as the graphic rating method; Hayes & Patterson, 1921). VAS presents participants with a horizontal line, usually with anchors at the left and right edges of the scale, representing disagreement and agreement with a statement. Subsequently, participants indicate their selection by clicking on the line. Their response is then mapped to the nearest pixel on the line, thereby enabling the distinction of values that exceed the resolution of discrete rating scales. Even though the general principle of selecting a response to a one-dimensional question with a fixed minimum and maximum value is similar between continuous and discrete rating scales, the two types of scales are generally not interchangeable (Kliger et al., 2015; Price et al., 2012).

A plethora of methodologies, such as item response theory, have been formulated to extract continuous variables from discrete rating scales (Henninger & Meiser, 2020; Masters, 1982). Similar methodologies have also been proposed for responses from continuous rating scales (see Molenaar et al., 2022 for a summary of models) and there is an ongoing debate on how to best analyze data from continuous rating scales (Heller et al., 2016). The present work, however, focuses on a comparison of the measurement error associated with different continuous rating scales without the implementation of additional modeling procedures.

A substantial body of research has demonstrated the efficacy of VAS in acquiring measurements of high quality, with the scale being interval-based (Hofmans & Theuns, 2008). Reips and Funke (2008) asked participants to indicate different percentages on a line, showing that participants could map the inquired values with high accuracy onto the provided scale. Due to its measurement properties, VAS has been frequently utilized in medical and pain research (Alghadir et al., 2018; Bijur et al., 2001; Price et al., 2012; Tiplady et al., 1998) where obtaining high-accuracy ratings is particularly important. The hypothesis that VAS ratings are on a linear scale has been confirmed for both mild to moderate pain ratings (Myles et al., 1999) and severe pain ratings (Myles & Urquhart, 2005). The large body of research in that field testifies to the high efficacy of VAS in producing precise measurements. Conversely, the implementation of discrete rating scales in survey research can introduce a bias in responses due to the need for respondents to select an option that best aligns with their intended response, particularly when the intended response is not adequately represented by the available response categories (Joyce et al., 1975). Furthermore, ratings provided with VAS appear to be unaffected by anchor bias (Hofmans & Theuns, 2008; Krabbe et al., 2006), and there are indications that VAS ratings may be less affected by mode differences than discrete rating scales (Gerich, 2007). Moreover, Funke and Reips (2012) showed that higher construct correlations could be obtained with VAS than with 5-point discrete rating scales without resulting in different means and dropout rates. Concurrent findings were reported by Kuhlmann et al. in two different studies (2016, 2017). Finally, VAS appears to reduce measurement error in comparison with discrete rating scales (Alghadir et al., 2018).

In summary, a substantial body of research has demonstrated the superior precision and accuracy of VAS in comparison to discrete rating scales. However, it is imperative to acknowledge that not all continuous rating scales are equivalent to VAS. Visual analogue scales should not be confused with slider scales (Reips, 2021). Even though both types of scales are continuous and may appear strikingly similar, they are not equivalent in terms of their functionality (Funke & Reips, 2007; Funke et al., 2011). VAS initially displays an empty line, prompting respondents to mark and select their response. In contrast, slider scales feature a marker such as a thumb that is frequently set at the extremes or the center of the response scale.

Shortly after Hayes and Patterson (1921), Freyd (1923) developed the graphic rating scale, a continuous measurement tool where respondents placed a checkmark at any point on an unnumbered line to indicate their rating level, with researchers then manually measuring the distance from the edge to the marked point using a stencil. These early graphic scales were more akin to VAS than slider scales and designed to capture the underlying continuum of subjective experiences—such as pain, mood, or performance—that could not be adequately represented by discrete categorical responses.

The transition to online continuous scales (in VAS or slider format) occurred with the advent of web-based survey technology, which eliminated the tedious manual measurements formerly required when graphic scales were presented in paper-and-pencil format. Continuous scales in online surveys are interactive elements that require respondents to click on a line (VAS) or drag a marker along a line (slider) to select an answer, offering a digital replica of Freyd’s original graphic rating concept. Slider scales first had to be programmed for the online environment, but since HTML5 was officially released as a World Wide Web Consortium (W3C) recommendation (finalized standard), there is now a slider as new input type. However, research comparing online slider scales to traditional radio-button formats has revealed significant usability challenges. Funke et al. (2011) found that slider scales led to substantially higher break-off rates (odds ratio = 6.9) and response times in web surveys, with problems particularly prevalent among participants with lower educational levels, suggesting that the slider format requires more cognitive load or prior technical knowledge. Slider functionality like dragging sometimes requires programming components that may not work in some browsers or devices. Despite these challenges, slider scales and VAS continue to be used in web surveys due to their perceived advantages in reducing survey fatigue through more engaging, interactive experiences and their ability to provide finer gradations along a numerical continuum.

As noted, slider scales feature a marker, such as a thumb, which must then be dragged to a position that indicates the respondent’s response. This is achieved by first continuously holding the thumb, dragging it to the desired location, and then releasing it. This method of selecting a response is more complex than the one-click method used in VAS. Furthermore, if the slider is initialized at a valid location, it becomes impossible to distinguish between a valid response and a nonresponse to an item (Funke, 2016; Funke & Reips, 2007). Moreover, the occurrence of nonresponses may even be directly affected by the starting position in combination with the truly intended response (Buskirk, 2015). Initializing the slider thumb at a specific location may bias responses to this location by anchoring (Funke & Reips, 2007; Toepoel & Funke, 2018). A number of studies have indicated that slider scales are often accompanied by various additional issues. Funke et al (2011) found higher dropout rates and response times under slider scales than with discrete response scales, which was amplified among respondents with lower levels of formal education. Funke (2016) compared VAS, slider scales, and discrete response scales, replicating the findings of Funke et al. (2011) and further demonstrating that VAS did not share the negative side effects of slider scales. The extant literature has demonstrated that slider scales are associated with a higher rate of user disengagement. In contrast, VAS has not exhibited these disadvantages. To the best of our knowledge, however, no studies have directly compared VAS with slider scales regarding their accuracy or, conversely, the measurement errors associated with their respective formats. The goal of the present study is to close this gap by assessing whether VAS ratings are superior to those of slider scales.

We conducted a replication of Reips and Funke (2008) to identify the continuous rating scale that would yield the lowest measurement error. In their study, Reips and Funke tasked participants with rating the location of 26 percentage values on a horizontal line ranging from 0 to 100%. The original study investigated variants of VAS that differed with respect to the length (operationalized via the number of pixels the VAS line was composed of) and the presentation of values (percentages or ratios). The results of the study showed that VAS ratings were on a linear scale for all conditions, with percentages being more straightforward to rate than ratios. Consequently, in the present study, we only administered the ratings in the format of percentages. In addition, the present investigation encompassed not only VAS but also an array of slider scales that are depicted in Fig. 1. The primary objective of our investigation was to assess the differences between VAS and slider scales in general. To control for potential effects of the initialization of the slider thumb and its appearance, additional variants of slider scales were introduced into the study. Depending on the experimental condition, slider thumbs were initialized in the center, the left edge, or the right edge of the line used for indicating the ratings. These slider conditions were designed such that the slider thumb and the horizontal line would mirror the appearance of VAS upon selection of a first response. Furthermore, a single slider condition was incorporated where the slider was chosen according to the default behavior of the HTML slider without any modifications. The default slider had a large slider handle positioned centrally within the scale (see bottom of Fig. 1). The rationale behind this additional condition was to ascertain the measurement error associated with the slider scale that is most readily implementable in online studies, because it is part of the Hypertext Transfer Protocol (“HTTP,” Wikipedia, 2026). The sole modification implemented for all slider conditions entailed the disabling of the click functionality, thereby restricting participants to dragging the slider to the intended location. This was done to determine the impact of utilizing VAS with the click functionality as opposed to the slider functionality.

**Fig. 1:**
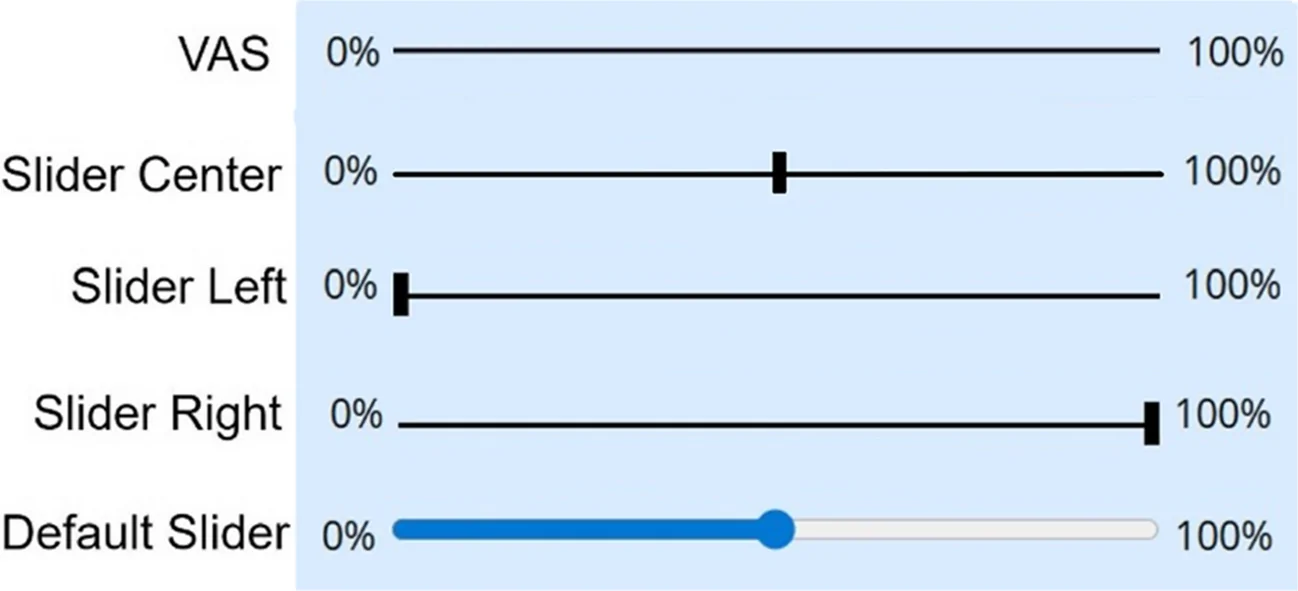
Rating scales by experimental condition at initialization. *Note*. The different scales were presented depending on the experimental condition. Upon selection of a response under VAS, a vertical line appeared at the location of the selected response with the same appearance as the first three slider scale conditions, i.e., with a width of 6 pixels

## Hypothesis

- H1: We expected that VAS would obtain a lower measurement error than the slider scale conditions because previous research revealed difficulties and lower engagement with slider scales (Funke, 2016; Funke & Reips, 2007; Funke et al., 2011).
- H2: We expected the default slider without any modifications to yield a higher measurement error than the other slider conditions due to the large thumb that we surmised would make selecting the appropriate location on the rating scale more difficult.

## Method

### Design

The present study was conducted as a between-subjects design with the factor response scale with the following conditions: VAS, slider initialized at the center, slider initialized at the left extreme, slider initialized at the right extreme, and the default HTML 5 slider initialized at the center without any modification to its appearance. Participants were randomly assigned to conditions at the beginning of the study. In each condition, participants had to provide 26 ratings where percentages would be located on a scale ranging from 0 to 100%. The absolute distance to the true value was evaluated for each response as the dependent variable. There were two counterbalanced orders in which the percentages were presented, as in Reips and Funke (2008).

### Material and procedure

The study was programmed and conducted on Wextor (https://wextor.eu; Reips & Neuhaus, 2002). On the first page, participants were informed about their rights, the use of their data, and the purpose of the study. Participants were also asked whether they intended to take part in the study in a serious manner (Aust et al., 2013; Reips, 2009). After providing informed consent, participants were required to provide demographic information regarding their gender and age on the second page. On this page, they were also informed that the experimental task would require them to decide where a particular value would lie on a scale either by clicking on the scale or by dragging a slider, depending on the condition. If the participants’ device had a resolution that was not wide enough to show the rating task, a warning message appeared instructing them to switch to landscape mode, if they used a smartphone for the study. Subsequently, the rating task commenced on the following page. As we replicated Reips and Funke (2008), we strictly adhered to their material. A total of 13 distinct percentages had to be identified on a scale from 0 to 100%. Each percentage occurred once in each of the two blocks A and B (see Table 1). The order of values within blocks was reversed, with the constraint that each block of percentages began with the value 50%. Whether participants first completed block A or B was determined randomly. Each percentage rating was presented on a separate page. Participants had to click the “Next” button to confirm their response and proceed to the next page. This gave them the opportunity to adjust their response as often as they wanted if they felt it was inaccurate. At the top of each page of the rating task, participants were reminded of the instruction in their condition, “Please select the position on the scale corresponding to [target]% by [dragging the slider/clicking the line].” Pointer events on the slider scale were disabled by setting the CSS property pointer-events to none, while the slider thumb remained draggable by setting pointer-events: auto to the thumb element. Information regarding the device type and size of the browser screen was extracted automatically using Wextor (Reips & Neuhaus, 2002).

**Table 1:** Percentage values displayed to participants

Percentages													
A	50	75	10	33	80	95	25	67	40	5	60	90	20
B	50	20	90	60	5	40	67	25	95	80	33	10	75

Table adapted from Reips and Funke (2008)

Each rating was conducted on a scale with a length of 300 pixels, irrespective of the specific condition.^1^ For the purpose of analysis, all scales were mapped to a range from 0 to 100%, thus reflecting the percentages of the task. With the exception of the default slider, all slider conditions were designed to mirror the appearance of VAS, thereby enhancing the comparability of the conditions (see Fig. 1). The width of the vertical line that appeared under VAS to mark a response, as well as the slider thumb of the first three slider conditions, was set to 6 pixels. The default slider exhibited a thicker handle and a filled meter extending from the left extreme of the scale to the handle. To ascertain the external validity of the default slider, no modifications were made to its appearance.

After completion of the rating task, participants were queried about the usability of the scales on the following two pages. The first of these pages asked participants, on a discrete scale, how accurate they thought their responses were utilizing the respective scale of the rating task, from 1 (*not at all accurate*) to 7 (*very accurate*). The second question on this page asked participants how much they enjoyed providing their response in the rating task on a scale from 1 (*not at all*) to 7 (*very much*). To ensure comparability of results across conditions, discrete response scales were employed for these questions, thereby ensuring that all participants evaluated the perceived accuracy and the enjoyment of utilizing the scales in the same well-known format. On the second page, regarding concerns of usability, we asked participants whether they found it necessary to zoom out or scroll sideways to view the entire scale in the rating task, and whether they used any aids, such as a ruler, to complete the rating task. In cases where aids were utilized, the participants were asked to specify the nature of the aids. Participants were assured that their responses to this question would not affect their participation or their remuneration, but rather would contribute to improving the quality of the results. On the final page, participants were thanked for their participation and debriefed about the purpose of the study. The median completion time for the study was 8 min, 59 s. Information regarding the device type and size of the browser was extracted automatically using Wextor (https://wextor.eu; Reips & Neuhaus, 2002).

### Statistical power and sample characteristics

We conducted an a priori power analysis with G*Power 3.1.9.7 (Faul et al., 2009). As we expected that the measurement error would be lower with VAS than with slider scales (H1), we computed the required sample size for an independent-samples *t*-test with a moderate effect size of *d* = 0.50 and an allocation ratio of 4:1 to the experimental conditions, as there were four slider scale conditions and one VAS condition. Based on this analysis, we concluded that we would need to recruit 216 participants to obtain power of .90. Participants were recruited via Prolific (https://www.prolific.com/). A total of 246 participants who signed the consent form began the study with 223 participants completing the study, whereby complete participation was defined as having at most four missing ratings in the rating task. As recommend by Reips et al. (2025) for dealing with dropout in online studies, we investigated whether missing values differed by experimental condition. No clear pattern emerged, as data from only a few participants had to be excluded due to an excess of missing values (between three and six people per condition). Data from a total of 222 participants were included in the analysis, after excluding data from one participant who indicated that they did not intend to take part in a serious manner. According to their self-report, the final sample consisted of 105 men and 117 women. Participants’ average reported age was 45 (*SD* = 13) years. The distribution of participants to experimental conditions followed approximately equal proportions, as can be seen in Table 2. To ensure that the allocation of participants to conditions did not differ from a uniform distribution, we computed a chi-squared test that revealed no systematic imbalance in the assigned conditions, χ(1) = 1.83, *p* = .767. For their participation, participants were rewarded with an equivalent of £6 per hour.

**Table 2:** Absolute error by response scale

	VAS (*n* = 45)	Slider center (*n* = 38)	Slider left (*n* = 48)	Slider right (*n* = 42)	Default slider (*n* = 49)
*M*	2.72	3.35	3.80	3.54	3.88
*SD*	0.89	1.80	1.72	2.27	1.99

VAS = visual analogue scale

### Statistical analyses

We used the R 4.4.2 (R Core Team, 2024) environment for statistical computing for conducting all statistical analyses. Additionally, we utilized the packages ggplot2 3.5.1 (Wickham, 2016), tidyverse 2.0.0 (Wickham et al., 2019), ggpubr 0.6.0 (Kassambara, 2020), effsize 0.8.1 (Torchiano, 2020), afex 1.4–1 (Singmann et al., 2021), and papaja 0.1.3 (Aust & Barth, 2022) for analyzing and plotting the results. All analyses were conducted using an alpha level of .05, where applicable.

## Results

### Analysis of raw errors

**Fig. 2:**
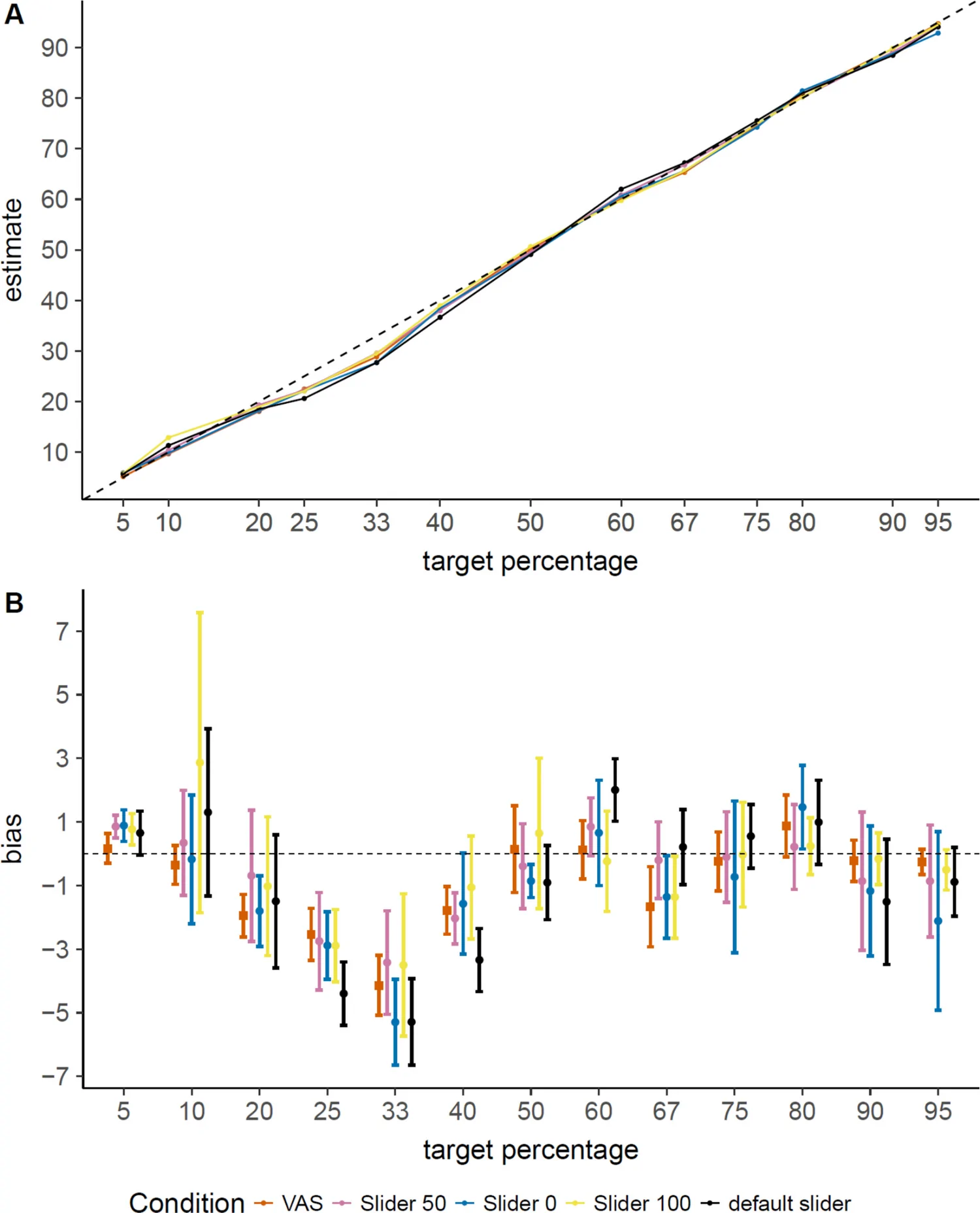
Bias in estimating the true value for VAS and slider scale conditions. *Note*. **A** Representation of true value by the participants’ estimated value. **B** Representation of the same data as in **A** but represented as the bias defined as the estimated value by the participants minus the true values. VAS is represented by squares and slider scales by dots. The error bars represent 95% confidence intervals

In the first step of the analysis, we computed the observed error in responses by subtracting the true value in the rating task from the indicated response. Responses belonging to the same percentage (two each) were averaged and plotted in Fig. 2. This analysis served to evaluate the proximity of our findings to those of the original study by Reips and Funke (2008). Overall, participants managed to identify the true value on the scale with considerable accuracy. However, it appeared that, consistent with Reips and Funke, participants tended to underestimate values between 10 and 50%, especially for 25% and 33%, thereby underscoring the replicability of the findings by the original authors.

### Analysis of absolute error

The analysis of absolute errors confirmed the findings in the previous section (see Fig. 2) that participants managed overall to locate the prompted percentages on the scale. For this analysis, we computed for each participant the average absolute error across the mean absolute biases for each percentage value. The mean and standard deviation of this metric can be found in Table 2. We did not observe any apparent differences between the types of slider scale conditions regarding their measurement error as computed with a two-factor analysis of variance (ANOVA) with the factors response scale and the counterbalancing factor, *F*(3, 169) = 0.56, *p* = .645, ${\eta}_{G}^{2}$ = .01. The main effect of counterbalancing and the interaction with the rating scale were not significant, *F*(1, 169) = 0.02, *p* = .897, ${\eta}_{G}^{2}$ < .01 and *F*(3, 169) = 1.71, *p* = .168, ${\eta}_{G}^{2}$ = .03, respectively. Consequently, the slider scale conditions were aggregated to a single group for the following analyses unless otherwise stated.

When comparing the absolute error under VAS (see Table 2) with the error under the slider scale conditions (*n* = 177, *M* = 3.66, *SD* = 1.95), a two factor ANOVA with the factors response scale and counterbalancing revealed that VAS responses were more accurate, *F*(1, 218) = 9.39, *p* = .002, ${\eta}_{G}^{2}$ = .04, which is consistent with H1. The effects of counterbalancing and the interaction between the response scale and the counterbalancing were not significant, *F*(1, 218) = 0.44, *p* = .506, ${\eta}_{G}^{2}$ < .01, and *F*(1, 218) = 0.26, *p* = .613, ${\eta}_{G}^{2}$ < .01, respectively.^2^

Contrary to H2, the absolute error was not significantly higher under the default slider (*M* = 3.88, *SD* = 1.99) compared to all other slider conditions (*M* = 3.58, *SD* = 1.93), *F*(1, 173) = 0.72, *p* = .398, ${\eta}_{G}^{2}$ < .01. The effects of counterbalancing and the interaction between the response scale and the counterbalancing factor were not significant, *F*(1, 173) = 0.82, *p* = .366, ${\eta}_{G}^{2}$ < .01 and *F*(1, 173) = 3.19, *p* = .076, ${\eta}_{G}^{2}$= .02, respectively. However, upon examination of the results depicted in Fig. 2B, it becomes evident that the discrepancy between the true value and the indicated response was most pronounced in the default slider condition among all conditions for values of 40% and 60%. This phenomenon can be attributed to the default slider’s initialization with a large thumb, which might have resulted in its displacement to the left beyond the 40% mark and to the right beyond the 60% mark.

### Analysis of covariates

A subsequent analysis revealed that the measurement error was generally higher among smartphone users (*n* = 23, *M* = 4.77, *SD* = 2.54) than users of a computer or laptop (*n* = 199, *M* = 3.32, *SD* = 1.67), *t*(24.24) = 2.66, *p* = .014, *d* = 0.82. This may be because smartphone users must use their fingers and not a computer mouse to indicate their responses, which might have led to the lower response accuracy because of the width of the finger (Park & Xiong, 2021). However, even when repeating the analysis of absolute errors only with participants who used a desktop computer or a laptop, the absolute error was still lower under VAS (*n* = 41, *M* = 2.64, *SD* = 0.87) than with the slider scale conditions (*n* = 158, *M* = 3.50, *SD* = 1.78), *F*(1, 195) = 8.44, *p* = .004, ${\eta}_{G}^{2}$ = .04. Again, there was no effect of counterbalancing, *F*(1, 195) = 0.09, *p* = .764, ${\eta}_{G}^{2}$ < .01, and no interaction between the response scale and the counterbalancing factor, *F*(1, 195) = 0.11, *p* = .736, ${\eta}_{G}^{2}$ < .01.

Furthermore, an exploratory analysis was conducted to determine whether browser window size could account for the observed results (analysis of covariance, ANCOVA). To this end, we again compared the absolute error under VAS with the slider conditions while controlling for the width (*M* = 1510.26, *SD* = 500.96) and the height of the participants’ browser screen (*M* = 945.95, *SD* = 169.29). Results were robust to this additional analysis, demonstrating the superiority of VAS over slider scales, *F*(1, 216) = 9.86, *p* = .002, ${\eta}_{G}^{2}$ = .04. The effect of counterbalancing and its interaction with the response scale were not significant, *F*(1, 216) = 0.51, *p* = .475, ${\eta}_{G}^{2}$ < .01 and *F*(1, 216) = 0.22, *p* = .640, ${\eta}_{G}^{2}$ < .01, respectively. Generally, participants with larger browser screen widths showed lower errors, *F*(1, 216) = 7.06, *p* = .008, ${\eta}_{G}^{2}$ = .03, whereas a similar result could not be observed for browser screen height, *F*(1, 216) = 0.40, *p* = .527, ${\eta}_{G}^{2} <$ .01.

### Analysis of consistency in responses

To assess the consistency of responses, we assessed the variability in absolute errors between and within participants. First, we compared the variance in the absolute error under VAS with the variance in the absolute error under slider scales (see Table 2), *F*(*df*_1_ = 176, *df*_2_ = 44) = 4.79, *p* < .001, showing that the variability in errors was reduced under VAS. This finding is substantiated by the reduced width of the confidence intervals of VAS ratings in Fig. 2B. We further examined each participant’s variability across all 26 absolute errors across measurements, as measured by the standard deviation. The resulting index of variability per person was then compared between VAS (*M* = 2.44, *SD* = 1.44) and slider scales (*M* = 4.34, *SD* = 4.36), *F*(1, 218) = 8.09, *p* = .005, ${\eta}_{G}^{2}$ = .04. This analysis showed that VAS even led to a lower variability in errors on an individual level when identifying the true values on a scale. Again, the effect of counterbalancing and the interaction between the response scale and the counterbalancing factor were not significant, *F*(1, 218) = 0.03, *p* = .858, ${\eta}_{G}^{2}$ < .01, and *F*(1, 218) = 0.02, *p* = .875, ${\eta}_{G}^{2}$ < .01, respectively. In summary, the present findings show that ratings utilizing VAS compared to slider scales are associated with a lower error, reduced variance in errors across participants, and reduced variance in errors on an individual level.

### Usability of rating scales

Finally, we investigated how accurate participants thought their responses were and how much they enjoyed the rating task. On average, participants thought that their responses were fairly accurate on a scale from 1 to 7 (*M* = 4.96, *SD* = 1.00). Additionally, we found that participants who indicated higher perceived accuracy of their responses in the rating task also produced lower errors in the rating task, *r*(220) = − .27, *p* < .001, indicating that participants had to some extent an idea of how well they performed in the rating task. However, there was no difference between VAS (*M* = 5.04, *SD* = 0.98) and slider scales (*M* = 4.94, *SD* = 1.01) in participants’ estimated accuracy of their own responses, *F*(1, 218) = 0.49, *p* = .486, ${\eta}_{G}^{2}$ < .01. The counterbalancing factor and the interaction between the employed scale and the counterbalancing factor did not affect this result, *F*(1, 218) = 0.21, *p* = .648, ${\eta}_{G}^{2}$ < .01 and *F*(1, 218) = 0.45, *p* = .503, ${\eta}_{G}^{2}$ < .01, respectively.

Furthermore, there was no correlation between the enjoyment of the rating task (*M* = 5.16, *SD* = 1.81) and the absolute error obtained in the rating task, *r*(220) = − .05, *p* = .488. There was also no difference in the enjoyment of the rating task between VAS (*M* = 5.29, *SD* = 1.80) and the slider scales (*M* = 5.12, *SD* = 1.82), *F*(1, 218) = 0.43, *p* = .513, ${\eta}_{G}^{2}$ < .01. There was no effect of counterbalancing, *F*(1, 218) = 0.09, *p* = .767, ${\eta}_{G}^{2}$ < .01, and the interaction between the response scale and the counterbalancing factor, *F*(1, 218) = 2.39, *p* = .123, ${\eta}_{G}^{2}$ = .01, did not affect results.

Ironically, the estimated accuracy of the responses and the enjoyment when performing the rating task were assessed using a discrete and not a continuous response scale to ensure that all participants answered the usability items in a format they were equally familiar with in this experiment. It is possible that the preference for VAS as indicated by the higher engagement than for slider scales in previous studies (Funke, 2016) might have been obfuscated by the less accurate discrete response format. Finally, none of the participants indicated having used any additional aids during the rating task, and only six participants indicated that they had to zoom out or scroll sideways during the rating task to see the full scale.

## Discussion

In the present study, we compared two continuous rating scales with different response formats: VAS and slider scales. The findings indicate that VAS ratings are associated with a lower measurement error and show reduced variability in errors between and within participants relative to slider scales. Furthermore, our results cannot be explained by accounting for additional covariates, such as the device type and the browser window size. Contrary to our hypothesis, the default HTML slider did not consistently demonstrate a higher measurement error than the other slider scale conditions that mirrored the appearance of VAS. We also did not find any apparent adverse effects of slider initialization (left-center-right) on the measurement error obtained in the rating task. Our findings indicate that researchers who intend to utilize continuous rating scales should consider the use of VAS over slider scales, given the lower measurement error.

Previous research has argued for the more frequent application of VAS over discrete rating scales due to its superior measurement properties (Alghadir et al., 2018; Funke & Reips, 2012; Kuhlmann et al., 2017; Reips & Funke, 2008). In accordance with the findings that VAS facilitates the acquisition of high-quality measurements, the present investigation has demonstrated that VAS also appears to outperform continuous rating scales, which require the user to drag a slider to provide a response, thereby supplementing the existing body of research regarding problems with slider scales such as lower engagement and higher dropout (Funke, 2016; Funke et al., 2011). It is important to note that the largest biases to the correct percentages were observed for percentages between 20 and 40% (see Fig. 2), replicating the findings by Reips and Funke (2008). However, these comparatively larger biases do not appear to be the cause of the observed discrepancy between VAS and the slider scale conditions. For instance, VAS ratings also demonstrate enhanced precision for extreme percentage ratings, such as 5% or 95%, while exhibiting considerably narrower confidence intervals for the majority of ratings. Our results may be particularly relevant in research domains where it is crucial to obtain measurements with the highest accuracy possible. A notable example of this is research focused on the study of pain (Alghadir et al., 2018; Heller et al., 2016; Myles & Urquhart, 2005; Myles et al., 1999; Price & Harkins, 1987).

The findings of the present study show the superiority of VAS over slider scales with regard to the absolute measurement error, which corresponds to an average of 0.94%. In absolute terms, this effect is relatively small. At the same time, we see that this difference between VAS and slider scales is of medium size according to the conventional interpretation of effect sizes proposed by Cohen (2013). It can be argued that, for researchers and practitioners deciding between different response scales, the more crucial question is whether to opt for a discrete or a continuous rating scale. This is in view of the substantial body of research demonstrating the higher precision of continuous rating over discrete rating for continuous constructs (see Alghadir et al., 2018; Funke & Reips, 2012; Kuhlmann et al., 2016, 2017). However, we would argue that even minor reductions in the observed measurement error are a worthwhile consideration, given the pervasiveness of continuous ratings, for instance in fields such as medicine and pain research (e.g., Heller et al., 2016; Kliger et al., 2015). And the meaning of effect sizes depends on context; in the case of Kuhlmann et al. (2016), for example, the otherwise small effect of difference in mortality meant several lives that likely were saved.

To enable a direct comparison of the different functionalities in the investigated rating scales, the click functionality in slider scales was deactivated. The rationale for this procedure was to ascertain which mode of responding, clicking versus sliding, would yield the lowest measurement error in the responses. When implementing a slider scale in HTML, the clicking functionality is activated by default. We would surmise that the measurement error in such scales lies between the error obtained with VAS ratings and that with slider scales of the present investigation. However, given that no direct comparison was conducted between VAS and slider scales with click functionality, the possibility remains that slider scales with click functionality may in fact outperform VAS with regard to measurement error (even though the literature generally points to low performance of slider scales). It is recommended that future research address this knowledge gap. This research may potentially result in the formulation of recommendations concerning the utilization of slider scales; whether they should be relinquished in favor of VAS, or alternatively, how participants should be optimally instructed to effectively use slider scales. The present study demonstrates that the click functionality generally leads to lower levels of error than when using only the sliding functionality. Consequently, researchers intending to employ slider scales are strongly advised to always incorporate the click functionality.

The objective of our study was to ascertain the discrepancy between VAS and slider scales with high power. Our analysis further demonstrated that no discernible disparities were observed among the various slider scale variants. In light of these findings, it can be concluded that VAS appears to be associated with lower absolute error levels than slider scales. However, it is important to consider the possibility that certain types of slider scales may not differ significantly from VAS. Future research should address this gap by specifically comparing slider scales—such as the slider that was initialized in the center of the scale and that had the lowest relative absolute error of all slider scales—with VAS to follow up on this question.

A potential limitation of the present study is that the majority of participants used a desktop computer or laptop to complete the study, leaving only a few participants using a smartphone. Given the prevalence of smartphone usage, future studies may benefit from exploring the replicability of these findings among smartphone users. A recent study by Park and Xiong (2021) reached the conclusion that slider scales outperform traditional VAS among smartphone users, a finding that is at odds with the results by Funke (2016). However, the two studies employed different implementations of VAS and slider scales. In addition, the study by Park and Xiong only recruited a sample of 28 students, who used the same smartphone under strictly controlled experimental conditions. In contrast, the study by Funke recruited a large sample of over 1,000 participants from a diverse background. A direct replication of the present study, employing the methodology of Reips and Funke (2008) in a large sample of smartphone users, would provide further insight into these conflicting findings.

A recent study has shown that the precision of VAS could be further enhanced by incorporating additional tick marks along the scale to denote percentages, e.g., in steps of 20% (García-Pérez & Alcalá-Quintana, 2023). The incorporation of additional marks into the VAS format was found to result in a reduction in both absolute errors and the standard deviation of absolute errors. This pattern of reduced measurement error as well as reduced variability in the errors was also observed in the present study when comparing VAS with slider scales. It would be worthwhile to investigate whether the superiority of VAS over slider scales remains consistent when supplementary visual aids are provided on the scale to assist in calibrating responses.

An advantage (which on the surface may be seen as a shortcoming) of the present study is that participants were asked to provide responses to percentage ratings rather than to commonly employed survey items, such as judgment tasks or personality inventories. Nevertheless, we would argue that the present results bear relevance for studies that extend beyond the direct estimation of percentage values on a rating scale. The present replication of Reips and Funke (2008) focuses on a specific aspect of the question–answer process, as delineated in Schwarz and Oyserman (2001), namely the mapping of an internal response to the available response scale. This process occurs in response to any question that requires responses expressed on a rating scale with a predefined format. Directly asking for percentage ratings has the advantage of mitigating the systematic and unsystematic errors that might otherwise obfuscate potential discrepancies in measurement error between response scales, such as understanding, assessing, and formulating an internal response to the question. However, to improve the generalizability and the scope of the present work, future research should investigate under what circumstances continuous rating scales, such as VAS, contribute to improved data quality. For instance, a study might ask that participants provide ratings for stimuli, such as the concreteness ratings of multiword expressions (Muraki et al., 2023) or subjective ratings of faces (Ma et al., 2015). The investigation could then proceed to examine the reduction in the number of absolute ratings necessary to achieve stable estimates of ratings, in comparison with discrete and other continuous rating scales.

In summary, the present findings clearly indicate that ratings obtained with VAS and slider scales are not interchangeable. VAS ratings result in lower measurement error as well as lower variability in errors. Reduced variability in errors was present on both a group level and an individual level, showing that VAS ratings may facilitate the detection of effects present across groups of participants and changes on an individual level. Finally, researchers aiming to improve the quality of their data might also consider using additional tick marks when using continuous rating scales (García-Pérez & Alcalá-Quintana, 2023) or consider providing numerical feedback to participants for them to calibrate their responses (Couper et al., 2006). However, it should be noted that there is some evidence that additional visual marks may systematically bias response distributions to the included marks (Matejka et al., 2016).

## Data Availability

All data are available at OSF under https://osf.io/wn7e8/overview?view_only=7e845e9449ad45a0bffca4d1bbc0cb4a.

## Footnotes

1 A technical error occurred during the mapping process, resulting in the mapping of ratings in the slider conditions to a scale ranging from 0 to 200 and from 0 to 300 under VAS. It is noteworthy that the length of the scale, which was set at 300 pixels, remained constant for all conditions. In order to guarantee that VAS would not possess an artificial advantage over the slider conditions with regard to measurement error, VAS values were mapped to an equivalent range of values and rounded so that it would have the same number of 201 distinct possible values. The findings remained unaltered by this modification, as the maximum enhancement in the precision of VAS relative to the other conditions due to this error could not surpass 0.167%. This is considerably lower than the effects reported in this paper.

2 Based on a comment by the first reviewer, we repeated our analysis using only those observations that showed an absolute bias of less than 20%. Results show that our analysis is robust to this exclusion, *F*(1, 218) = 5.50, *p* = .020, ${\eta}_{G}^{2}$ = .02, with the absolute error being lower under VAS (*M* = 2.65, *SD* = 0.79) than under the slider scale conditions (*M* = 3.08, *SD* = 1.06). Effects of counterbalancing and the interaction of the scale with the counterbalancing factor again did not reach statistical significance, *F*(1, 218) = 1.71, *p* = .192, ${\eta}_{G}^{2}$= .01 and *F*(1, 218) = 2.34, *p* = .128, ${\eta}_{G}^{2}$= .01, respectively.

## Code availability

Analysis code is available at OSF under https://osf.io/wn7e8/overview?view_only=7e845e9449ad45a0bffca4d1bbc0cb4a.
